# Fungal Ferromanganese Mineralisation in Cretaceous Dinosaur Bones from the Gobi Desert, Mongolia

**DOI:** 10.1371/journal.pone.0146293

**Published:** 2016-02-10

**Authors:** Krzysztof Owocki, Barbara Kremer, Beata Wrzosek, Agata Królikowska, Józef Kaźmierczak

**Affiliations:** 1 Institute of Paleobiology, Polish Academy of Sciences, Warsaw, Poland; 2 Faculty of Chemistry, Warsaw University, Warsaw, Poland; The University of Wisconsin - Madison, UNITED STATES

## Abstract

Well-preserved mycelia of fungal- or saprolegnia-like biota mineralised by ferromanganese oxides were found for the first time in long bones of Late Cretaceous dinosaurs from the Gobi Desert (Nemegt Valley, Mongolia). The mycelia formed a biofilm on the wall of the bone marrow cavity and penetrated the osteon channels of the nearby bone tissue. Optical microscopy, Raman, SEM/EDS, SEM/BSE, electron microprobe and cathodoluminescence analyses revealed that the mineralisation of the mycelia proceeded in two stages. The first stage was early post-mortem mineralisation of the hyphae by Fe/Mn-oxide coatings and microconcretions. Probably this proceeded in a mildly acidic to circumneutral environment, predominantly due to heterotrophic bacteria degrading the mycelial necromass and liberating Fe and Mn sorbed by the mycelia during its lifetime. The second stage of mineralisation, which proceeded much later following the final burial of the bones in an alkaline environment, resulted from the massive precipitation of calcite and occasionally barite on the iron/manganese-oxide-coated mycelia. The mineral phases produced by fungal biofilms colonising the interiors of decaying dinosaur bones not only enhance the preservation (fossilisation) of fungal remains but can also be used as indicators of the geochemistry of the dinosaur burial sites.

## Introduction

Microbial alteration is an important but still poorly understood pathway for bone degradation [1]. Fungi and fungi-like saprolegnia (water moulds) are ubiquitous saprophytes widely recognised in the archeological record as bone-degrading organisms which dissolve the bone matrix, producing characteristic branching tunnels [2]. Fungi form mats and, in combination with bacteria, can form biofilms enabling the precipitation of minerals in environments often considered unfavourable for mineralisation [3, 4]. In contrast to the well-understood biomineralisation phenomena observed in recent fungi and fungi-bacteria associations, there are only a few studies concerned with fungal or generally microbial biomineralisation in fossil bones and its influence on bone fossilisation and preservation [5, 6, 7, 8].

Although there is an extensive fossil record of fungi [9, 10, 11] extending back to the Precambrian [12, 13], the younger fossil record is sparse and limited largely to fungal remains preserved in amber [14, 15, 16, 17]. Well-preserved specimens have been described from the Paleozoic, including exceptionally well-fossilised Devonian fungi from the Rhynie chert [18, 19, 20, 21].

The aim of this work is to present the first known case of mineralised fungal or saprolegnia-like mycelia preserved in dinosaur bones exemplified in samples from the Cretaceous Nemegt Formation (Gobi Desert, Mongolia). Following fungal remnants of coprolites from the Lameta Formation (India) [22] and a *Penicillium*-like fungus from Early Cretaceous turtle eggs described from the Langtoutang Formation (China) [23], this is the third example of fossil mycelia associated with vertebrate fossils from Mesozoic strata, and the first finding in the archaeological record of fungal borings and hyphae occuring together in fossil bones. The mycelia described here are the oldest fossil fungal- or saprolegnia-like remains identified from bones to date.

## Geographic and Geological Location

**Fig 1:**
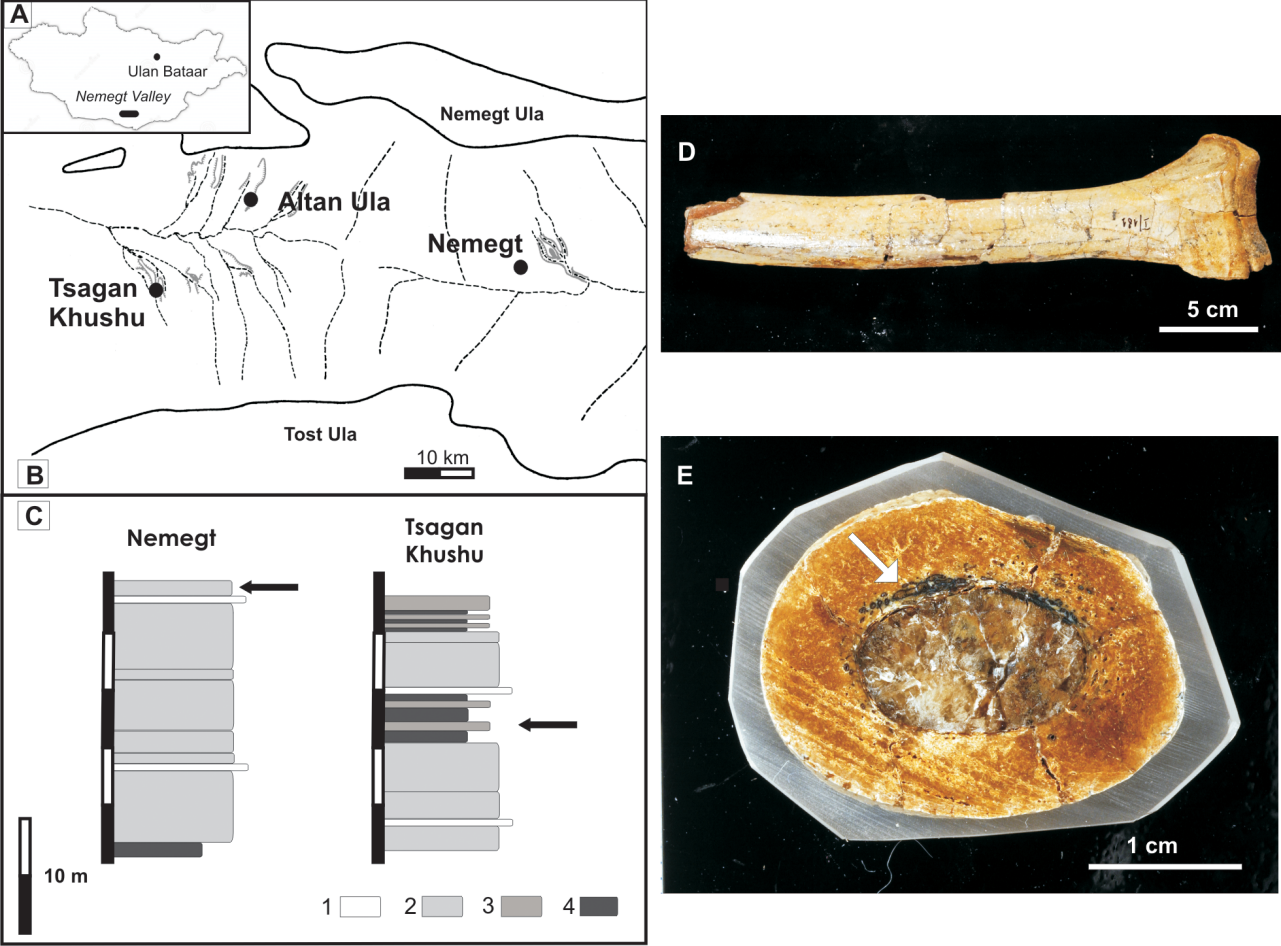
Geographical and geological location of the collecting sites. (A) Location of the Nemegt Valley on a map of Mongolia (B) Map of the Nemegt Valley with the locations of the Altan Ula, Nemegt and Tsagan Khushu sites (from Kremer et al., 2012, modified) (C) Lithological profiles with the indicated position of the examined specimens from the Nemegt and Tsagan Khushu sites (after Gradziński et al, 1977, modified) (D) Fragment of a metatarsal; specimen ZPAL MgD I/181. E: Polished transversal sections of dinosaur bones from specimen ZPAL MgD I/alt. The position of mineralised fungal or saprolegnia mycelia is indicated by an arrow. Note the middle of the marrow canal filled with transparent calcite and canal walls encrusted with oxides.

The examined material was found in the Upper Nemegt beds (Nemegt Formation) by a Polish-Mongolian expedition during a field campaign in 1965 in the Nemegt Basin of the Trans-Altai Gobi in Mongolia (Fig 1). The age of the Nemegt Formation is estimated as late Campanian-Early Maastrichtian, and its exposures are famous for being among the most fossiliferous in the world [24, 25, 26, 27]. The Nemegt formation consists of fining-upward successions of channel fillings (sandstones and mudstones) limited by erosional cuts and intraformational conglomerates interrupted by flood plain deposits. Common sedimentary features of the Nemegt beds include scoured surfaces and erosional channels filled with conglomerates and siliciclastic sediments with inclined stratification, large-scale cross stratification, climbing ripples, fining-upward cycles and other features indicative of a fluvial depositional environment [24, 28, 25]. Most of the bipedal dinosaur skeletons were characterised by an arrangement of elements corresponding to a vertical projection onto a horizontal surface [24] with tails usually strongly bent backwards, necks with head (if preserved) bent in the same way, and contracted hind limbs [24]. All of the analysed specimens belonged to *Gallimimus bullatus* and showed a high degree of articulation. Specimen ZPAL MgD I/8 from Nemegt is a fragmentary postcranial skeleton of subadult size. This specimen was discovered in the top part of the fining-upward sequence of oxbow lake infill, in fine-grained sands with mud intercalations and small-scale cross/wavy lamination. The studied specimen ZPAL MgD I/181, which derives from the Tsagan Khushu site (Nemegt Basin), is a fragmentary hind limb with the pelvis and the sacral portion of the vertebral column, found in flood plain deposits (a 2.5-m-thick layer of fine-grained sand with small-scale wavy lamination and deformational structures in contact with concretion-rich mudstone). Due to a lack of archival data, little is known about the single bone labelled ZPAL MgD I/alt, except that it was found at the Altan Ula II site by the 1964 Polish-Mongolian Expedition. The position of the examined specimens from the profiles at Tsagan Khushu and Nemegt is presented in Fig 1.

## Material and Methods

No permits were required for the described study, which complied with all relevant regulations. A set of longitudinal and transverse standard petrological polished thin sections about 30 μm thick was made from *Gallimimus bullatus* bones: the left femur of specimen ZPAL MgD I/8, (Nemegt), a metatarsal from specimen ZPAL MgD I/181 (Tsagan Khushu), and a phalanx from ZPAL MgD I/alt (Altan Ula).

Histological observations were made with an optical microscope (Nikon Eclipse LV100 POL). Opaque authigenic minerals were identified with a reflected-light microscope and a Phillips XL-20 scanning electron microscope equipped with an ECON 6 energy-dispersive (EDS) detector, a EDX-DX4i system, and an FEI backscatter electron (BSE) detector for compo or topo detection. This instrument was operated at an accelerated voltage of 25 kV, a beam current of 98‒103 nA, and a spot diameter of 3.5 μm. Additional chemical analyses were made using a Cameca SX 100 electron microprobe at the Joint-Institute Analytical Complex for Minerals and Synthetic Substances (University of Warsaw). The microprobe was used for carbon-coated polished thin sections under the following conditions: an accelerated voltage of 15 kV, a beam current of 20 nA and a beam spot of 1.5 μm. Well-defined minerals and synthetic phases were used as standards. The peak counting times were 10 s for major and 20 s for minor elements. At these durations, the average detection limits were: 0.028‒0.046 wt% for Ca, Na, K, Al, Si, Sr, P, Mg and Cl; 0.071‒0.074 wt% for Fe and S; 0.116 wt% for Mn and Ba; 0.319 wt% for F and Zn.

SEM observations and SEM/EDS analyses were performed on small (15×15 mm) polished thin sections; mineralised mycelia were etched for 5‒10 s with 5% formic acid, rinsed with distilled water and sputtered with platinum. Cathodoluminescence analyses were performed at the NanoFun laboratories (Cathodoluminescence Microscopy Laboratory) at the Institute of Paleobiology, Polish Academy of Sciences, on a Lumic HC5-LM cathodoluminescence microscope. The vacuum (approximately <10‒5 mbar) was produced by a high-vacuum turbomolecular pumping system. The HC5-LM was integrated with a Princeton Instruments (Acton Series SP-2356) high-efficiency UV-VIS spectroscopic system and a Kappa DX 40–285 CL air-cooled colour CCD camera. The microscope used a hot cathode (tungsten filament).

The specimens examined in this study are housed in the Institute of Paleobiology of the Polish Academy of Sciences in Warsaw (abbreviated ZPAL).

### Raman data collection

Raman measurements were performed at the Laboratory of Intermolecular Interactions, Faculty of Chemistry, University of Warsaw. Raman point mapping was carried out by means of a LabRAM HR800 (HORIBA Jobin Yvon) spectrometer, coupled with an Olympus BX61 confocal microscope, provided with a 100× objective, using a diode-pumped, frequency-doubled Nd:YAG laser (532 nm, Pmax = 100mW on the head). Spectra were collected in a backscattering configuration, with a confocal pinhole size set at 200 μm, using a holographic grating with 600 grooves/mm. The spectrometer was equipped with a Peltier-cooled CCD detector (1024 × 256 pixels). The calibration standard used was the well-known 520 cm^−1^ Raman band of a silicon chip.

Point Raman mapping was performed on a sample area 32.5 μm × 31.3 μm, proceeding with a 1-μm lateral increment step. Here, analysed spectra contributing to Raman maps were collected with the power of the laser beam on the sample in a range of 1 mW. The acquired spectra ranged from 100 to 1800 cm^−1^. Five accumulations with an acquisition time of 3 s were collected for each point spectrum. Single spectra covering the 100–4000 cm^−1^ spectral range (not shown here) were also collected in addition to the mapping experiment. The Raman spectra and maps were obtained and analysed using LabSpec5 software.

Hierarchical cluster analysis (HCA) is a powerful method for examining large sets of data for common characteristics. HCA was performed using D-value distances and Ward’s clustering algorithm for the merging of spectra.

D-values used for distance matrix calculation are defined as follows:

$$d_{jk}=(1-r_{jk})\cdot 1000$$

where *r*_*jk*_ is known as Pearson’s correlation coefficient [29, 30]:

$$r_{jk}=\frac{\left(\sum_{i=1}^{n}x_{ji}\cdot x_{ki}\right)-n\cdot {\bar{x}}_{j}\cdot {\bar{x}}_{k}}{\sqrt{\left(\sum_{i=1}^{n}x_{ji}^{2}-n\;{\bar{x}}_{j}^{2}\right)}\cdot \left(\sum_{i=1}^{n}x_{ki}^{2}-n\;{\bar{x}}_{k}^{2}\right)}$$

A distance matrix is a symmetrical matrix of the size *n* × *n*, where *n* is the number of analysed spectra; it is a dissimilarity matrix with zero diagonal elements, such that the off-diagonal *ij*-th terms represent the distance between or degree of dissimilarity of the *i*-th and *j*-th objects (here: spectra). The obtained interspectral distances provide information on the similarity of the spectra and enable them to be merged in groups of spectra, called clusters, according to their homogeneity. Ward’s method [31], known to generate small (dense) clusters, was employed for hierarchical clustering of the similarity measures:

$$d_{i,jk}=\left[\frac{1}{n_{i}+n_{j}+n_{k}}\right]\cdot \left[(n_{j}+n_{i})d_{ji}+(n_{k}+n_{i})d_{ki}-n_{i}\cdot d_{jk}\right]$$

D-value distances and Ward’s algorithm implemented in CytoSpec software (v 1.4.02) were used to create HCA imaging. Raw multispectral Raman data were used without any pre-processing. Two spectral regions were analysed for HCA purposes: 100‒800 and 801‒1800 cm^−1^. Only the reassembled colour-encoded HCA image and obtained mean cluster spectra (no dendrogram) from CytoSpec are presented. CytoSpec software (v 1.4.02) was made available courtesy of Professor Małgorzata Barańska, Faculty of Chemistry, Jagiellonian University.

## Observations

### Bone microstructure and composition

**Fig 2:**
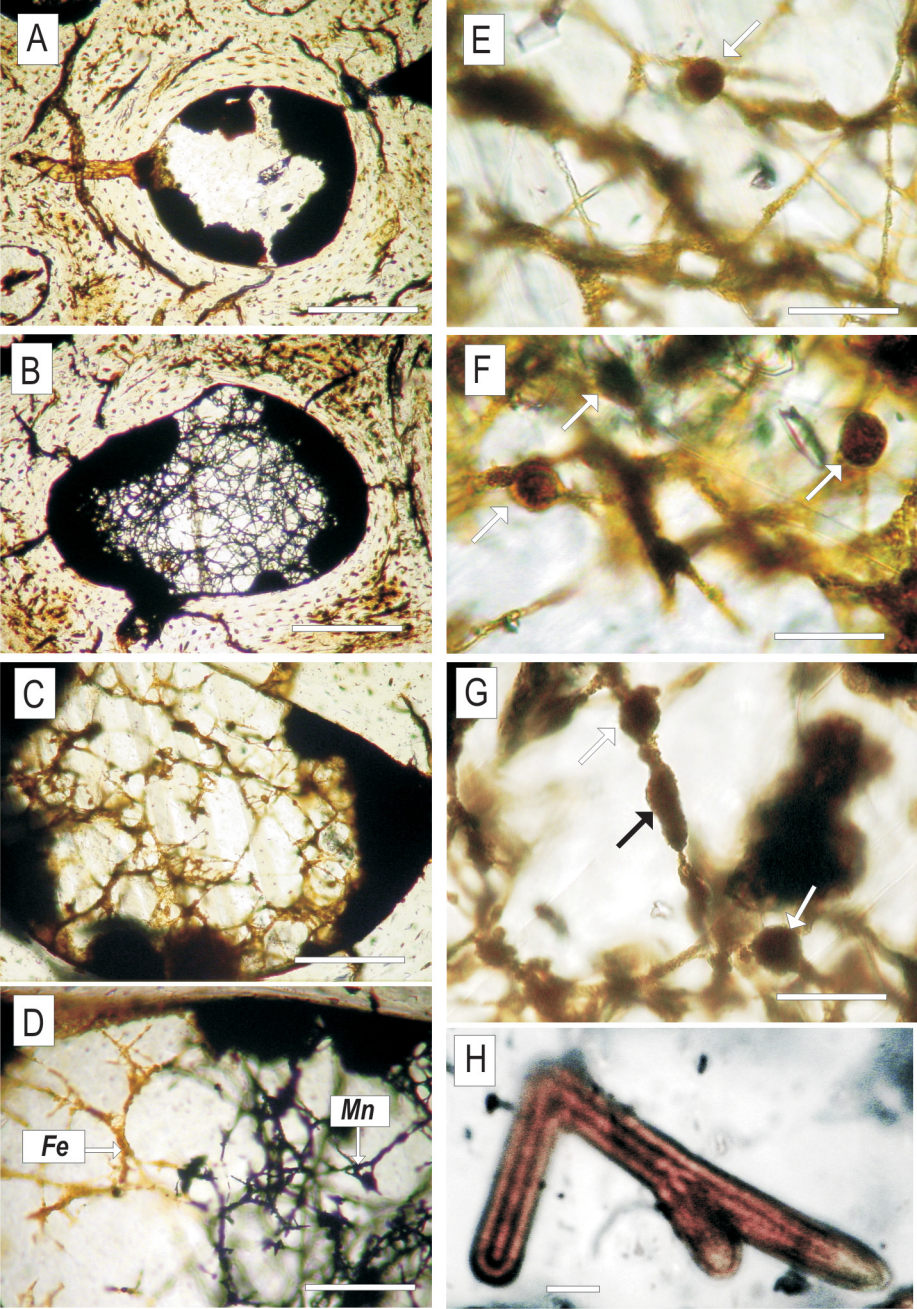
Transmitted light images of mycelia preserved inside dinosaur bone. (A‒C) Bifurcating translucent hyphae embedded in calcite inside the marrow cavity of ZPAL MgD I/alt bone (D) A network of hyphae embedded in calcite filling a bone void; note the changing mode of hyphae mineralisation, from black manganese oxides to brown iron oxides (E‒G) Network of hyphae with putative asexual reproductive bodies (?spores) indicated with arrows (H) Fragment of iron-manganese-oxide-permineralised mycelium showing the distinctly siphonous organisation of the branching hyphae. Scale bars: (A‒C) 100μm (D) 50 μm (E-G) 20 μm (H 5) μm.

SEM and microscopic studies revealed that all of the examined bones show excellent preservation of histological features, such as canalliculi and osteocyte lacunae with a clear plywood structure visible in transmitted light (Fig 2A and 2B). In most of the specimens, radial cracks and flaked inner circumferential layers of the compact bone indicate the swelling of collagen during early diagenesis due to undergoing hydrolysis in an water-rich environment [32, 33].

**Fig 3:**
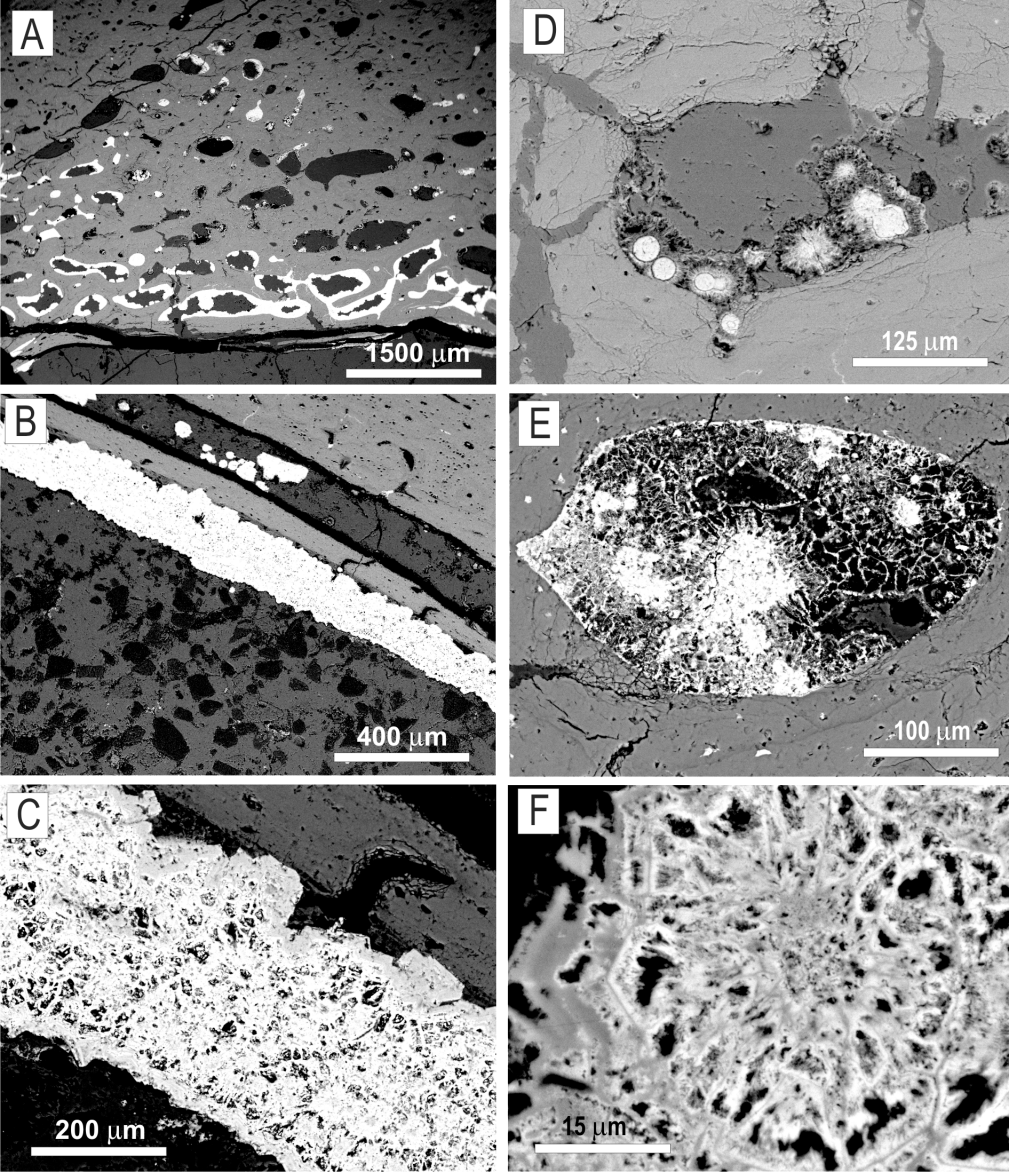
SEM/BSE images of mycelia mineralised with Fe/Mn oxides and calcite with an occasional admixture of barite. The mycelia (white) occur either as dense biofilms composed of subglobular hyphal aggregates covering the wall of the marrow cavity (A‒C) or as individual sunflower-like aggregates and networks of hyphae filling the resorption canals inside the compact bone tissue (D‒F). Specimen ZPAL MgD I/8 (B, C, F), specimen ZPAL MgD I/alt (A, D, E).

**Fig 4:**
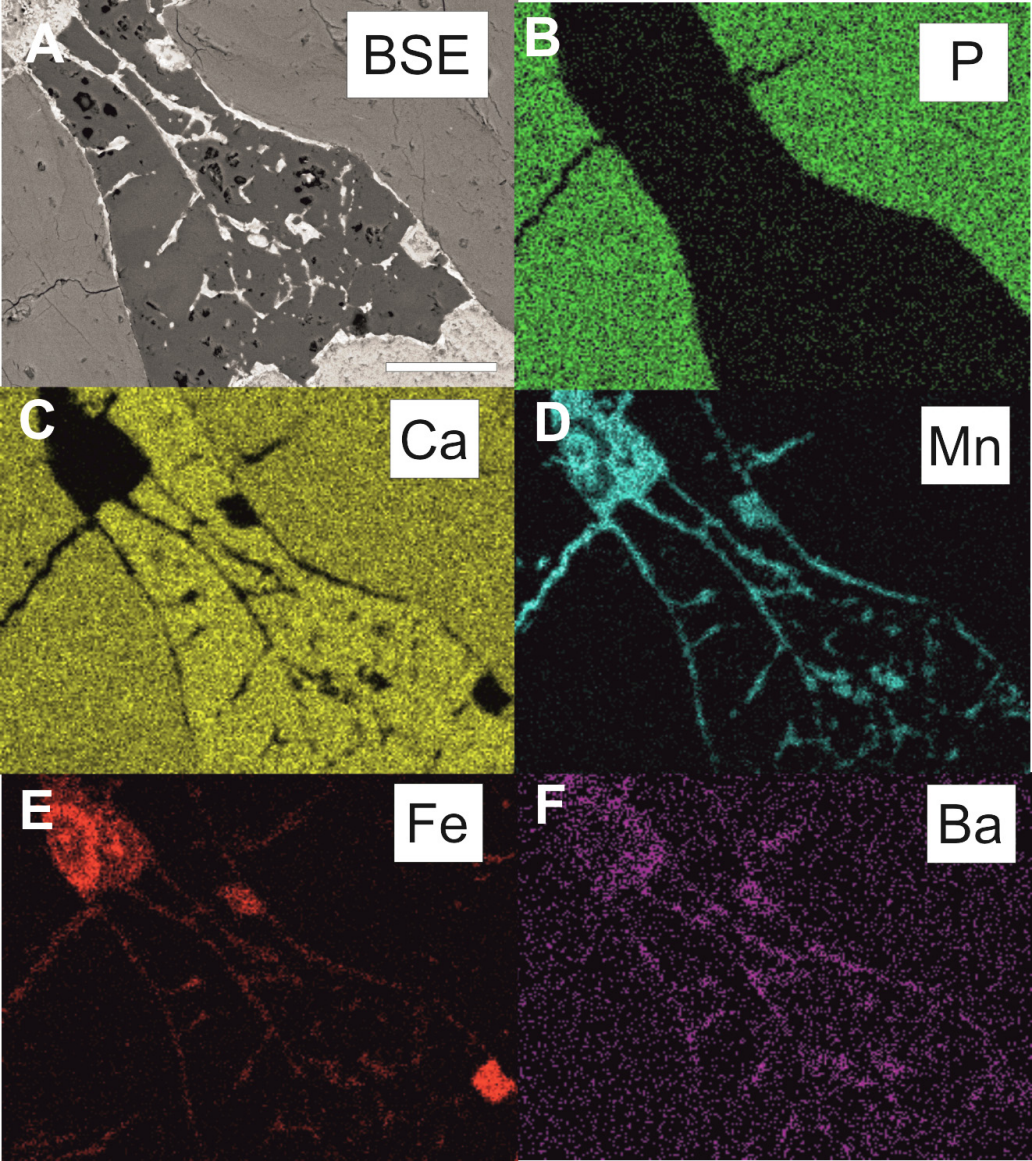
SEM/EDS elemental maps of hyphae from a resorption canal inside the dinosaur bone. Specimen ZPAL MgD I/alt from the Altan Ula site. Scale bar for all images corresponds to 100 μm.

Examined bones from specimens ZPAL MgD I/8 and ZPAL MgD I/181 are characterised by a fibrolamellar structure with a secondary haversian system partially developed in more endostellar parts of compact bone. The bone in specimen ZPAL MgD I/alt is characterised by a well-developed haversian system with primary osteons and pervasive radial cracks indicative of water uptake and swelling of bone collagen [33]. In the case of specimen ZPAL MgD I/8, many osteocyte lacunae, vascular canals and diagenetic radial cracks are filled by oxides forming casts, which provide strong contrast to surrounding fossil bone apatite in BSE images (Fig 3). In the specimens from Nemegt and Tsagan Khushu, most of the histological cavities in the compact bone are large (over 100 μm wide) and empty; the diagenetic cracks in specimen ZPAL MgD I/8 are filled with calcite, barite and ferromanganese oxides. All examined specimens possess a medullar cavity filled with millimetre-sized sparry calcite crystals. In the specimen from Nemegt, micritic calcite mixed with oxide biofilm and rare subhedral crystals of barite form a mineralisation zone separating bone from the sparry calcite filling the medullar cavity. Microprobe analyses and EDS spectra (Fig 4) showed that the bones are composed of secondary carbonate/fluoride apatite (francolite).

### Biofilm and Fungal Tunneling

**Fig 5:**
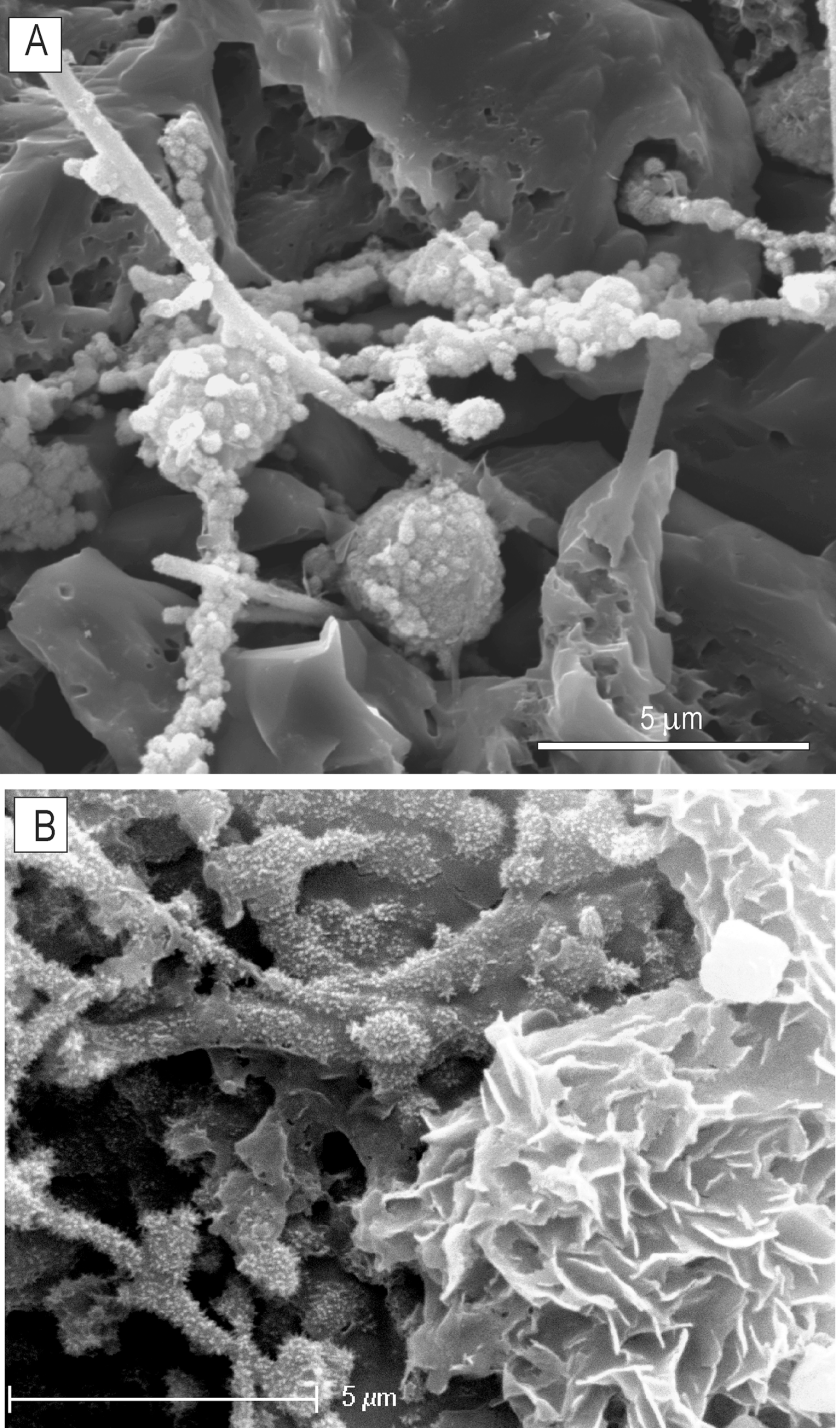
SEM images of a mineralised mycelium exposed with formic acid etching from polished thin section ZPAL MgD I/8 bone. (A) Hyphae and putative asexual reproductive bodies (?spores) covered with Fe/Mn nanograins (B) A fragment of organically preserved hyphae (left) and a rosette-flower-like pattern of ferromanganese micronodules precipitated into the mycelium biomass (right).

**Fig 6:**
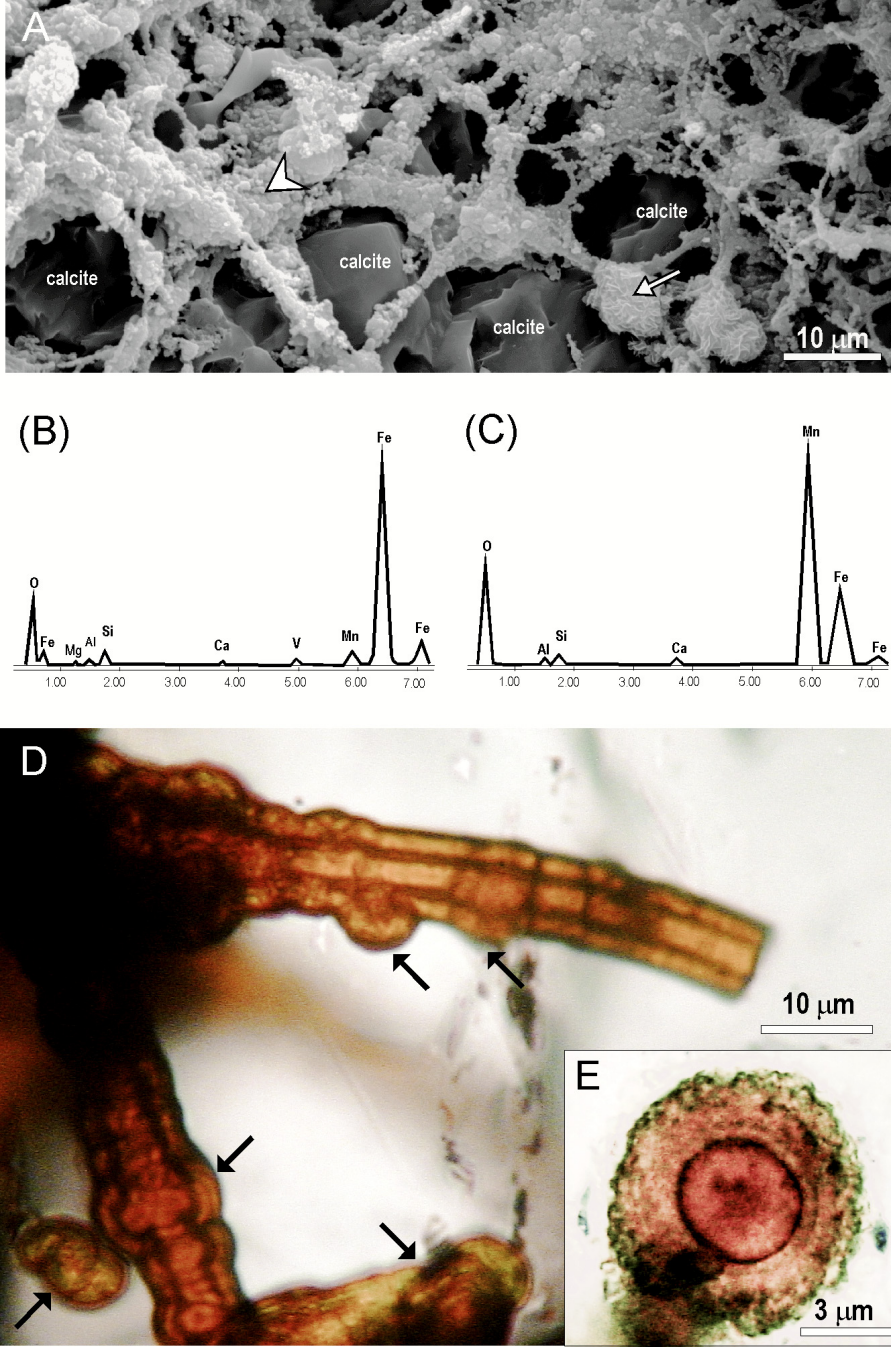
SEM images, SEM/EDS data and light micrographs of the mineralised mycelia. (A) SEM image of a mineralised mycelium from polished and formic-acid-etched thin section of ZPAL MgD I/8 bone. Note the reticulate pattern of hyphae covered by ferromanganese nanograins (arrowhead) and the mycelium associated with ferromanganese micronodules (arrow) (B) EDS spectrum of ferromanganese nanograins coating on hyphae indicated by arrowhead in (A) showing a high Fe signal (C) EDS spectrum from a mycelium-associated ferromanganese micronodules indicated by arrow in (A) showing a high Mn signal (D) Transmitted light micrograph of magnified hyphae showing sparse septation and ferromanganese micronodules precipitated on the surface (E) Highly magnified micrograph of a cross section of a ferromanganese micronodule (micro concretion) showing its concentric structure. Specimen ZPAL MgD I/181.

The walls of the medullar cavity of all examined specimens are coated with oxides which range from opaque to translucent in transmitted light (Fig 2). Translucent oxides are accumulations of ovoidal structures 10‒20 μm in diameter, similar to the ‘micro concretions’ described by Kremer et al. [34] from the tibia of a *Saurolophus* dinosaur from the Tsagan Khushu site (Figs 5 and 6). The thickness of oxide coatings varies from 50 μm in ZPAL MgD I/181 to over 2 mm in ZPAL MgD I/8. In specimens ZPAL MgD I/8 and ZPAL MgD I/181, the inner circumferential layer and adjacent secondary osteons are detached from compact bone; the resulting space is filled with micritic calcite partially mixed with biofilm oxides (Fig 3A‒3C).

Sparse microbial borings of the Wedl type 1 according to the classification of Hackett [35] (modified by Trueman and Martill [36]) penetrate the bone with a starting point at the oxide-bone contact (Fig 2A). These borings are characterised by a diameter of 15‒40 μm; those present in the large vascular channel in specimen ZPAL MgD I/181 show branching, while others connect oxides in neighbouring bone canals (ZPAL MgD I/alt) or form single tunnels in lamellar bone of the inner circumferential layer (ZPAL MgD I/8) (Figs 2A and 3C).

SEM investigations show that the microstructure of oxide coatings is complex, consisting of nanograins and rosette-like aggregates (Fig 3). Subunits 10‒35 μm in diameter can be distinguished, with a central oval area occupied by interconnecting struts and rods. Hundreds of filaments 1.5‒5μ wide show Y-branching and radiate from the central area to form a matted mass with filaments from adjacent centers. Both microprobe analyses and EDS spectra indicate that these biofilms consist of non-stoichiometric Fe oxides with the addition of Ca, Mg, Mn, Al, Si, P and V as minor elements (Table 1). However Mn-Ba oxides were identified in ZPAL MgD I/alt as a major constituent of all coatings in bone canals.

**Table 1:** Electron microprobe analyses of the Mn- and Fe-rich mineral phases of fossilised biofilm from the *Gallimimus* bones.

	ZPALMgDI/8	ZPALMgDI/8	ZPALMgDI/8	ZPALMgDI/8	ZPALMgDI/8	ZPALMgDI/8	ZPALMgDI/alt	ZPALMgDI/alt	ZPALMgDI/alt
**P**_**2**_**O**_**5**_	0.15	0.11	0.18	3.49	0.21	0.29	0.16	0.24	0.21
**K**_**2**_**O**	0.01	0.00	0.01	0.00	0.00	0.00	0.01	0.00	0.00
**CaO**	0.57	0.37	0.48	6.19	0.92	1.06	1.23	0.59	0.63
**Fe**_**2**_**O**_**3**_	82.78	80.06	85.30	69.78	91.90	76.49	22.70	76.12	87.46
**MnO**	0.97	0.91	1.04	0.87	0.93	2.32	45.63	1.31	1.81
**BaO**	0.00	0.25	0.00	0.00	0.00	0.16	10.70	0.11	0.11
**V**_**2**_**O**_**3**_	0.07	0.23	0.62	0.64	0.21	0.36	1.04	2.46	0.55
**Na**_**2**_**O**	0.00	0.00	0.00	0.00	0.00	0.00	0.21	0.00	0.00
**MgO**	1.07	1.62	0.75	0.94	0.25	0.29	0.38	1.09	0.41
**Al**_**2**_**O**_**3**_	0.49	0.25	0.38	1.79	0.16	1.87	0.83	1.46	0.37
**SiO**_**2**_	3.06	2.76	2.77	4.52	1.34	7.35	1.59	3.28	2.02
**sum**	89.16	86.54	91.51	88.22	95.92	90.18	84.59	86.67	93.58
**OH/CO**_**2**_	10.84	13.46	8.49	11.78	4.08	9.83	15.41	13.34	6.43

### Mycelia and Hyphae

The quality of morphological preservation of the mycelia and hyphae in the examined samples is similar to that of some specimens found inside amber of various geological ages, as described in the literature [15–17]. However, more details, like biochemical composition of the cell walls (chitin or cellulose) or characteristics of the reproductive structures are needed to make a trustful taxonomic identification. Therefore, not much can be said concerning the systematic position of the preserved fungal-like remains. On a morphological basis, the presence of sparsely septated and dichotomously branching hyphae and putative bodies resembling spores, along with the generally sunflower-like appearance of the mycelia, suggests similarity to true fungi or fungi–like saprolegniales (water moulds) known to be saprophytic forms colonising the dead bodies of macro organisms.

Mycelia are abundant in the cancellous canals of the examined samples and connect with the oxide coatings on the canal walls. Hyphae have smooth or nano-spiny walls (Figs 5B and 6D) and sparse septa (Fig 6D) and show bifurcated branching (Fig 2H). Some hyphae are characterised by irregular surfaces encrusted with oxide material, particularly in apical and branching parts. Most striking are sharp differences, clearly visible in transmitted light, in the composition of the oxide coatings. In a single hypha, a change from opaque Mn-oxides to transparent Fe-oxides can be easily traced (Fig 2D). An element distribution map was compiled using an EDS detector for a mycelium preserved inside the minor medullar cavity in specimen ZPAL MgD I/alt. Fig 4 shows that calcium phosphate is the component of the bone, while calcium carbonate (calcite) is the mineral phase filling the bone cavity, which confines fungal-like hyphae consisting of manganese oxide with various, mostly small, amounts of Fe and Ba.

In specimen ZPAL MgD I/181, mycelia are preserved in the medullar cavity and are adjacent to the inner circumferential lamellae of the compact bone. Hyphae are transparent, characterised by Y-branching and smooth walls. In both specimens, hyphae show excellent preservation in three dimensions and are embedded in clean transparent calcite filling the bone cavities. Their preservation mode is comparable to that of the Lower Cretaceous mycelia described from ambers of Northern Spain [15] and Myanmar [16].

The biofilm coating of the medullary wall in bone ZPAL MgD I/8 is very thick compared to the mycelia found in specimens from Altan Ula and Tsagan Khushu. Its bulky nature makes it opaque in transmitted light and massive in BSE and SE images from SEM (Fig 3). However, in ‘overpolished’ sections, its fibre structure, with characteristic branching typical of fungal hyphae, can be distinguished. SEM investigations of the acid-treated polished bone fragments show that the fungal structures consist of iron oxides forming a network of branching hyphae 1.5‒4 μm wide, with a ‘lumpy’ surface that is smooth in some areas. Attached to some hyphae are ovoid to subglobular, minutely roughened bodies, 8‒10 μm in diameter, resembling spores known to occur both in fungal and saprolegnia mycelia. An EDS spectrum collected from one of these structures indicates that their main constituent is manganese oxide.

### Raman identification of mineral and non-mineral compounds

**Fig 7:**
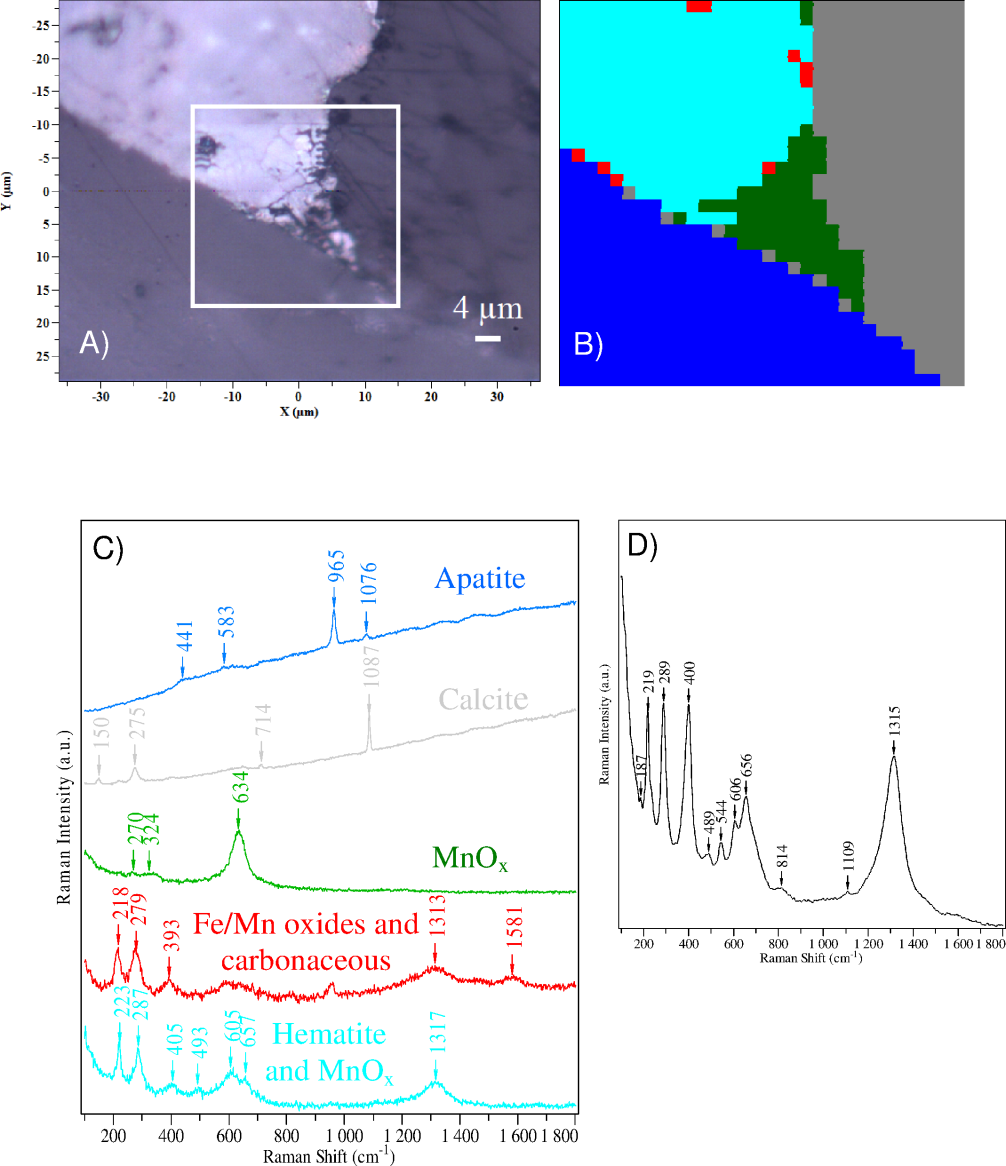
Raman spectral data collected from mineralised mycelium. (A) Microscopic image of the studied bone in reflected light; the frame marks the mapping areas from which Raman spectra were collected (B) Colour-coded Raman map of five clusters after hierarchical cluster analysis (HCA), based on spectral similarities for the bone section studied here, shown in (A). Colour codes are assigned to each of the five identified clusters (C) The representative cluster Raman spectra obtained from HCA. Colours represent individual cluster classes (D) A representative Raman spectrum collected from mineralised mycelium. Specimen ZPAL MgD I/alt.

The chemical composition and lateral distribution of the mycelium mineralisation products were examined by means of the Raman point mapping technique (ZPAL MgD I/alt). Several fungi objects were analysed, but only representative results are presented in Fig 7. An optical microscope image collected in reflected light for the selected location in bone tissue is shown in Fig 7A. The area subjected to Raman analysis is framed with a white rectangle, corresponding to the limits of the mapping grid. Multivariate methods of analysis are very useful for extracting small yet relevant differences in spectra collected from different regions of the sample and for the further construction of chemical images from these multispectral datasets. Hierarchical cluster analysis (HCA) was successfully performed in our previous work to validate the manual classification of variations in spectral features of individual Raman spectra from different regions of mineral microspheres formed during bone diagenesis [24].

The area selected for Raman mapping encompassed a fragment of bone, with its marrow cavity inhabited by fungi and mineralised by various mineral phases. The HCA map demonstrates the heterogeneity of components in the petrographic section and clearly shows the details of their lateral distribution (Fig 7B). The mean cluster spectra, indicated with the same colours and representing the spectral features characteristic of a given cluster, are given in Fig 7C. According to Raman mapping, the fungal mycelium is mostly composed of Fe and Mn oxides (shown in light blue, red and green in Fig 7C) and trace amounts of carbonaceous material (red). Calcite (grey) and apatite (dark blue) are also present in the HCA image (Fig 7B). The dark blue cluster spectrum (Fig 7C) can be unambiguously ascribed to the Raman signature of apatite [37]. The strongest band, at 965 cm^−1^, corresponds to the symmetric stretching vibration of PO_4_^3−^, while the bands between 400‒600 cm^−1^ are due to PO_4_ tetrahedra bending modes [37]. The apatite identified here can be classified as a diagenetically altered sample, as the full width at half maximum (FWHM) of the PO_4_^3−^ symmetric stretching vibration is less than 13 cm^−1^ (a FWHM of 11.8 cm^−1^ for the bioapatite studied here), while the band position is greater than 964.7 cm^−1^ (here: 965 cm^−1^). These two spectral parameters have been proposed as indicative of the diagenetic transformation of apatitic fossils, sufficient to alter the oxygen isotopic composition of lattice-bound phosphate [38]. The crystallinity index (CI_Raman_) of apatite can also be calculated using the definition given by Reynard et al. [39] and used by Pucéat et al., [40], where CI_Raman_ = 4.9/Γ is a ratio of the FWHM of the discussed PO_4_^3−^ symmetric stretching mode (Γ) in the sample and magmatic apatite as a reference. The value of CI_Raman_ for the sample examined here is around 0.42 (as Γ = 11.8), which indicates a low crystallinity value for the bioapatite.

The light blue trace in Fig 7C shows mostly the Raman bands characteristic for hematite around 225, 285, 405, 495, 605 and 1315 cm^−1^ [41, 42]. Their positions are downshifted and their relative intensities markedly changed compared to previously studied hematite dominated by mineral microspheres of microbial origin found in a bone of the same Late Cretaceous dinosaur [34]. This may confirm a different mineralisation pathway, related to the presence of fungi-like structures, for the bone regions studied here. Still, the spectral pattern presented as a light blue trace in Fig 7C shows some additional bands which cannot be assigned to hematite vibrational modes. The most intense appears around 655 cm^−1^, while another can also be distinguished around 545 cm^−1^ (see the spectrum in Fig 7D for a single Raman spectrum showing more details in this region). The former band can be very likely assigned to the vibrational mode of manganese oxides, whose presence has been already detected by SEM/EDS analyses. Unfortunately, the wavelength of the green excitation line (532 nm) used here is close to the energy of electronic transition in hematite [43]. Therefore, the Raman signal of hematite is selectively enhanced under our experimental conditions due to the Resonance Raman effect. On the other hand, Raman spectroscopy has been only moderately applied to the study of MnO_x_ materials [44, 45, 46, 47, 48] and bacterially produced manganese oxides [49, 50, 51]. In these studies, both visible and UV Raman were used to study manganese oxides. UV Raman turned out to be more suitable for studies of MnO_2_ materials because it absorbs visible light strongly, hence reducing Raman scattering [50]. The position of the Raman band around 655 cm^−1^ observed here may be indicative of the presence either of MnO_2_ [45, 50] or Mn_3_O_4_ [45]. However, even Raman spectra for the same lattice type of MnO_2_ (ramsdellite) excited with an identical laser line (514.5 nm) may differ in terms of peak positions and relative intensities [44, 45]. Bernard et al. [52] demonstrated that manganese oxides may be unstable upon illumination with a green laser (514.5 nm). This thermal effect induced by a laser resulted in the replacement of the major Raman bands at 650, 576, and 523 cm^−1^, typical for pyrolusite MnO_2_, with one broad signal centred at 634 cm^−1^. The cluster spectrum shown by the green curve in Fig 7C, characterised by a main band around 634 cm^−1^, strongly resembles these spectral features [52]. Apparently the reduction of MnO_2_/Mn_3_O_4_ by a laser beam occurs during Raman mapping experiments for the sample studied here in the region marked in green in the HCA image (Fig 7B). The cluster spectra depicted in light blue and green in Fig 7C are also quite different from the Raman signatures of birnessite- and todorokite-like manganese oxides, which, it has been proposed, are formed as bacterially produced manganese oxides [50] and biogenic manganese oxide mineral coatings in a subsurface granite environment [51].

An additional spectral feature visible around 544 cm^−1^ (Fig 7D) may also arise from MnO_x_ lattice vibrations [45]; however, it may also be indicative of goethite formation [42]. Further evidence for goethite presence in the studied section may be provided by the easily visible shift of the hematite band from 405 (the light blue trace in Fig 7C) to 393 cm^−1^ (the red trace in Fig 7C). It is difficult to decide whether this band shift is related to the presence of native goethite in the sample or an effect of local heating due to a relatively high level of local laser power [53]. The red cluster spectrum in Fig 7C also shows a weak band around 1580 cm^−1^, which can be ascribed to the G peak of carbonaceous material [54]. The hematite 2LO (longitudinal optical) mode around 1315 cm^−1^ is located in the spectral region of the disorder-related band (D peak) from *sp*^*2*^ carbonaceous materials; thus these two can easily be confused, as observed by Marshall and Marshall [55]. In this case, it is highly likely that the 2LO mode of hematite is obscuring the D peak of carbon. However, a red cluster spectrum is distinctive for only a few locations in the region presented here and was observed rather rarely for other analysed regions of the sample (not shown here). The last cluster spectrum (shown in grey in Fig 7C) shows Raman bands around 1085, 715, 275 and 150 cm^−1^. Their positions and relative intensities are in good agreement with the spectral pattern of calcite in the literature [56]. Vibrational modes at 1085 and 715 cm^−1^ correspond to symmetric stretching and in-plane bending, respectively, of the carbonate group, while the two peaks with low wave numbers are due to lattice modes [56]. Unfortunately, no Raman signature of barite was detected during a mapping experiment [57]. Still, the results of Raman mapping confirm two stages of mycelium mineralisation: the first by Fe/Mn oxides and the second occurring with a precipitation of calcite.

## Discussion

Traces of fungal activity in fossilised or archeological bones are usually associated with Wedl-type boring [35, 58, 59, 60, 61]. Such microbial borings occur in the vascular channel in the bone ZPAL MgD I/181 (Fig 2A and 2B) and a small number of unbranching tunnels were also found in lamellar bone in the inner circumferential layer in specimen ZPAL I/8. In the first case the channel is filled with opaque iron oxide, while some of the other tunnels are empty. In all examined specimens, the mycelia consist of either iron or manganese oxides and form a branching network of hyphae 2‒5μm wide. In the case of bone from specimen ZPAL MgD I/181, the branching hyphae are made of translucent hematite or a similar iron oxide phase. Hyphae in the specimen from Altan Ula are composed of hematite and opaque black non-stoichiometric manganese oxide, in which within one hyphae iron-bearing phase can be transformed into a manganese-rich phase (Fig 2D). Mycelia in this specimen sometimes form sunflower-like associations reaching nearly 70 μm in diameter and consisting of mainly iron oxides, as revealed by an elemental map (Fig 4). However, they differ morphologically from the sunflower-like framboids described by Sawłowicz [62].

The dinosaur bones from the Gobi desert were deposited in a fluvial-terrestrial environment in dry climatic conditions [24, 25, 26]. In recent river environments, skeletal remains are usually buried in sediments of the flood plain or in point bar and channel bar beds [63]. Nearly complete or partial articulated skeletons are usually the result of destruction of bodies of animals subject to longer transport and decomposition in which the skull and limbs are lost, the latter usually broken off at the joints [63, 64]. Fungi and water moulds are usually associated with terrestrial environments, where they play an important role in the mineralisation and cementation of sediments [3]. They are able to passively accumulate various mineral phases in the mucous envelopes around the thallus (e.g. [3, 65]); as a result mineral phases such as birnessite, ferrihydrite, goethite, hematite, todorokite, halloysite, and montmorillonite often crystallise around thalli ([3] and reference therein). These inlays and auto mineralisation can survive in the fossil record, as in the case of fungal hyphae and conidia found in fossilised eggs of turtles from the Chinese Liangtoutang Formation [23]. Santelli et al. [66] noted that Mn-oxidising bacteria are the main focus of recent studies, although in many terrestrial environments fungal Mn and Fe oxidation may also be important ([66] and reference therein; [65]). It has been shown that some fungi can promote the oxidation of Mn(II) to Mn(IV)O_2_ [67, 68, 69, 70, 71].This process in many cases is thought to be non-enzymatic, proceeding due to interaction with metabolic products and/or cellular components [72]. Fungi possess a sophisticated mechanism for the selective uptake and immobilisation of Fe and Mn [73, 74, 75, 76, 77]. They produce a number of organic substances such as lactate, oxalate, and glutamates, which acidify the surrounding medium and chelate the metal ions transported into cells and immobilised by polyphosphates. The examined fossilised fungi from dinosaur bones share a morphology similar to that of the recent Mn-oxidising fungi from the phylum Ascomycota, particularly the genera *Acremonium* and *Pyrenochaeta* [66]. These fungi precipitate nanocrystalline phyllomanganate onto their hyphae and asexual fruiting bodies. This amorphic phase is, in a way similar to that of birnessite or δMnO_2_ [78, 79, 80], abiotically transformed into more crystalline and thermodynamically stable phases such as todorokite or feithknechtite [78, 81]. Ascomycete fungi such as *Acremonium* [66] can produce a variety of iron and manganese phases depending on the pH of the surrounding medium and availability of metal ions. Ascomycete fungi are known from the fossil record mostly from amber findings [15, 16, 17] and were found as iron-oxide-mineralised hyphae and conidia from an Early Cretaceous turtle egg clutch [23].

**Fig 8:**
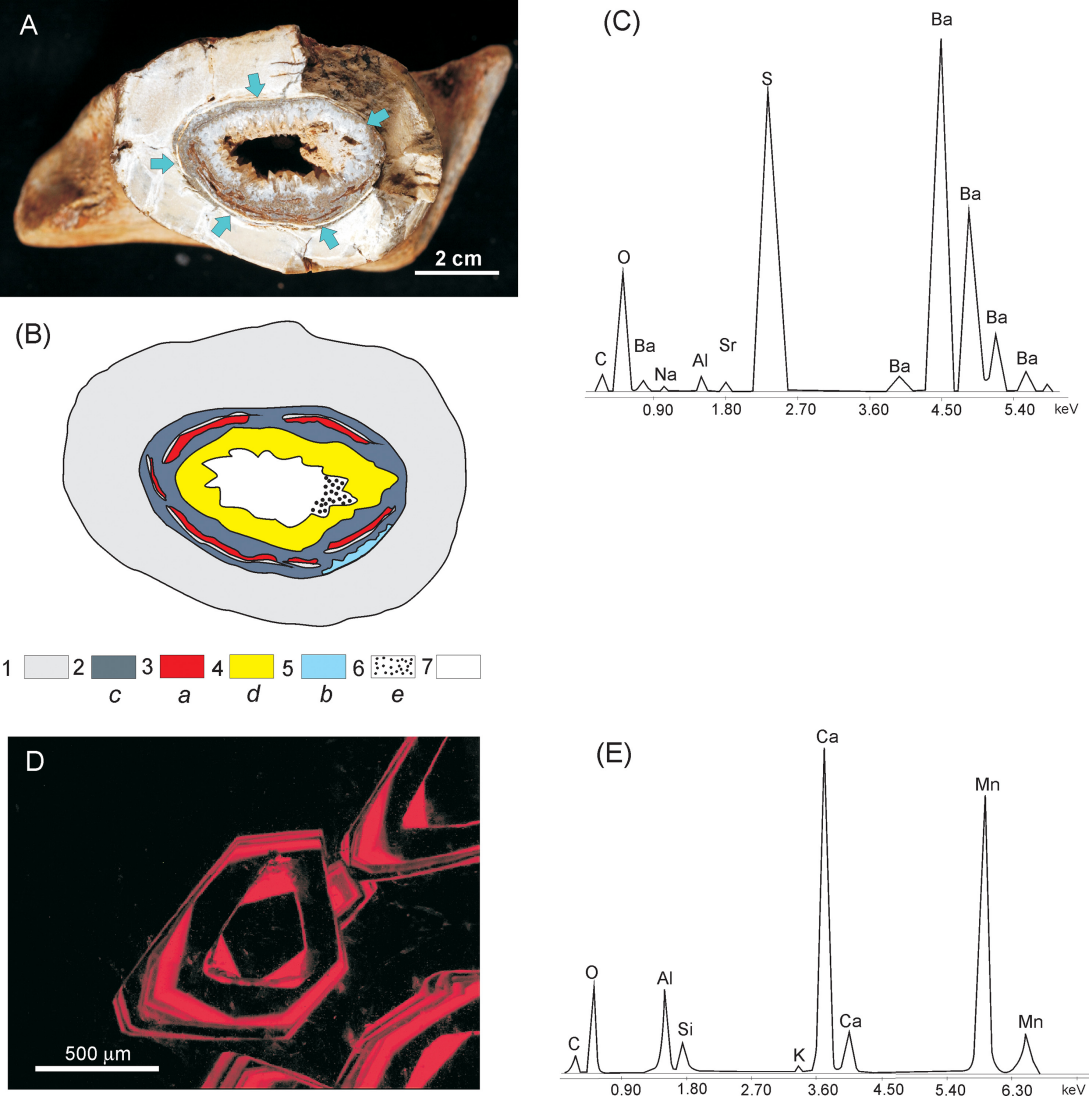
Successively precipitated mineral phases in marrow cavity. (A) Cross section of a femur bone (specimen ZPAL MgD I/8) showing a marrow cavity (delimited by arrows) filled almost entirely with largely microbially-mediated mineral succession (B) Schematic illustration of the mineral succession imaged in (A); 1 –massive bone, 2 –micritic calcite filling the marrow cavity colonised by fungi, 3 –mineralised fungal biofilm, 4 –abiotically precipitated crystalline calcite, 5 –barite, 6 –intrusion of clastic sediment, 7 –open space of the marrow cavity; lowercase letters indicate the sequence of precipitation of mineral phases from (a) to (e) (C) SEM/EDS spectrum documenting the presence of barite, precipitated in the marrow cavity as indicated in the mineral succession diagram in (B) (D) Cathodoluminescence image of banded calcite representing the later stage of mycelia mineralisation after the earlier diagenetically precipitated ferromanganese. (E) SEM/EDS spectrum documenting the presence of calcite.

The calcite crystallisation process appears to be crucial to understanding the different mode of preservation of fungal or fungal-like biofilms inside the dinosaur bones from the Nemegt Valley. The bones from the sites Tsagan Khushu and Altan Ula show, under cathodoluminescence, excellently preserved hyphae embedded in homogenous sparry calcite, suggesting rapid crystallisation under constant geochemical conditions in pore waters. However, in bone ZPAL MgD I/8 from Nemegt, the bulky mycelium is embedded (as revealed under cathodoluminescence) in a uniform, micritic calcite, while the later diagenetic sparry calcite is characterised by a distinct zonal structure, suggesting long-term crystallisation under cyclic changes in the composition of the diagenetic pore waters (Fig 8D).

Micritic calcite crystals or their mosaics filling bone voids are usually associated with crystallisation in the vadose zone, while sparry large crystals are formed in the zone of saturation (phreatic) (e.g. [82]). Barite indicates the presence of barium and sulphate ions in pore waters (Fig 8B and 8C). Cathodoluminescence studies of calcite crystal druse in the bone marrow cavity of the ZPAL MgD I/8 bone (Fig 8A) suggest cyclic formation of late diagenetic calcite expressed in the form of bands of varying manganese content (Fig 8D and 8E). However, in the bone ZPAL MgD I/alt from Altan Ula, enclosing well-preserved hyphae, the closing calcite biomineralisation is homogeneous, suggesting rapid crystallisation, or, less likely, long-term homogeneous hydrochemical conditions during precipitation. Stoops and Zavaleta [83] explain the formation of barite in soil environments with periodic groundwater sulphate saturation. Brock-Hon et al. [84] also suggest crystallisation of barite in moist soils with low pH or biomineralisation by oxidising bacteria. Contrary to the definition in the literature of calcite and barite as late diagenetic phases, these minerals are able to quickly fill a void in bone within one to two years, as was observed by Trueman et al. [85] in recent bones from the Amboseli National Park. In addition, actualistic taphonomical experiments [86, 87, 88, 89] show that decomposition of organic matter (in the case of a dinosaur, bone marrow would be most likely) can, in a few weeks, cause crystallisation of calcite. In addition, the collagen-degrading activity of microorganisms and soft tissues produces favourable geochemical gradients initiating rapid crystallisation of some mineral phases [90]. The presumably rapid crystallisation of calcite (Fig 8D and 8E) could indirectly contribute to the excellent preservation of hyphae in the studied dinosaur bones.

We found no similar detailed studies on ferromanganese mineralized fungal or fungi-like mycelia in the literature. An exception are findings of fossilised fungal remains in Oligocene/Miocene iron stromatolites of northwestern Germany with goethite, calcite and chalcedony/quartzite mineralized mycelia [91]. In this case the fungi colonized karstic hollow molds in Devonian limestone percolated by water rich in Fe^2+^ and colloidal Fe^3+^, a mineralization environment very different from that of the lacustrine-fluvial conditions characterizing the Mongolian dinosaur bones burial site. Excellent preservation of mycelia were several times described in amber of various age [14,15,16,17]. Amber is, however, a unique fossilization medium difficult to compare with that of dinosaur cadavers burial site.

## Conclusions

The organically preserved mycelia of fungi- or saprolegnia-like microbiota in the studied long bones of Late Cretaceous dinosaurs demonstrate the high fossilisation potential of early post-mortem ferromanganese mineralisation supported by immuration of the mycelia systems in subsequently precipitated calcite and barite (Fig 8). The observed two-step mineralisation of the fungal mycelia is a valuable microtaphonomical tool supporting macrotaphonomical field observations suggesting the Okavango delta as a modern analogue of the Nemegt Valley dinosaur burial environment [25, 26]. The presence of mineralised fungal or saprolegnia mycelia in the Gobi bones significantly enhances our understanding of the burial environment, which, based on the physicochemical conditions required for the precipitation of mineral phases, must have changed from semi-dry during the first ferromanganese stage of mineralisation to wet, but still oxygenated, in the late diagenetic calcite/barite stage.
